# Electroacupuncture for Menstrual‐Related Migraine: Protocol for a Multicenter Single‐Blinded Randomized Controlled Trial

**DOI:** 10.1155/prm/2200276

**Published:** 2026-07-20

**Authors:** Shihan Jiang, Qing Ye, Sichun Gu, Qiang Li, Qi Jin, Zhicheng Guo, Ping Yin, Lumin Liu

**Affiliations:** ^1^ Sleep Medicine Center, LongHua Hospital Shanghai University of Traditional Chinese Medicine, Shanghai, China, longhua.net; ^2^ Encephalopathy Department, LongHua Hospital Shanghai University of Traditional Chinese Medicine, Shanghai, China, longhua.net; ^3^ Neurological Department, Shanghai Gongli Hospital of Pudong New Area, Shanghai, China; ^4^ Acupuncture Department, Shanghai Municipal Hospital of Traditional Chinese Medicine, Shanghai University of Traditional Chinese Medicine, Shanghai, China, shutcm.edu.cn

**Keywords:** electroacupuncture, menstrual-related migraine, multicenter randomized controlled trial, protocol

## Abstract

**Importance:**

Menstrual‐related migraine (MRM) is a prevalent neurological condition affecting females, with prolonged duration, severe symptoms, and poor drug response, significantly impairing their quality of life. Electroacupuncture shows promise as a nonpharmacological intervention for migraine. However, there is a deficiency in high‐quality evidence‐based research assessing its efficacy in MRM. To address this gap, we present a detailed protocol to evaluate electroacupuncture’s therapeutic effects in MRM.

**Methods and Analysis:**

A total of 120 participants with MRM will be randomly divided into two groups (1:1): the electroacupuncture group and the sham electroacupuncture group. Participants will receive 30‐min treatments (electroacupuncture or sham) for 6 sessions every 4 weeks (18 sessions over 12 consecutive weeks). The primary outcome will be migraine days and attacks during Weeks 9–12 vs. baseline. Secondary outcomes (all vs. baseline unless specified) include the following: (1) frequency‐related: migraine days/attacks per 4 weeks (Weeks 1–24, excluding Weeks 9–12; and during menstrual/nonmenstrual periods). (2) Symptom characteristics: days/attacks with migraine‐associated symptoms and attack duration per 4 weeks. (3) Patient‐reported: visual analog scale (VAS), Migraine Disability Assessment (MIDAS) questionnaire, Insomnia Severity Index (ISI), Generalized Anxiety Disorder‐7 (GAD‐7), and Patient Health Questionnaire‐9 (PHQ‐9). (4) Others: treatment response rate, average dosage, and frequency of emergency drugs. All adverse events during the research will be documented. SAS 9.4 statistical software will be used for analyses.

**Trial and Dissemination:**

Ethical approval was obtained from the Medical Ethics Committee of LongHua Hospital Shanghai University of Traditional Chinese Medicine (No. 2024LCSY150), Shanghai Gongli Hospital of Pudong New Area (GLYY1s2024‐085), and Shanghai Municipal Hospital of Traditional Chinese Medicine (2025SHL‐KY‐60‐01), and the study was registered with the International Traditional Medicine Clinical Trials Registry. All participants who fulfill the inclusion criteria and consent to join the study will sign a written informed consent form before randomization.

**Trial Registration:** International Traditional Medicine Clinical Trials Registry: ITMCTR2024000795

## 1. Introduction

Migraine is a common neurological disease with an incidence rate ranging from 10% to 20% [1], which in females is two times that in males [2]. More than 50% of migraines are associated with menstruation in female patients [3]. Nearly 14% of female patients suffer from migraine only during the menstrual period, referred to as pure menstrual migraine (PMM), while 60% of them experience migraine both in menstrual and nonmenstrual periods, known as menstrual‐related migraine (MRM) [4]. Compared with PMM, there is usually no prodrome before an MRM attack, but it has a longer duration and more severe associated symptoms such as pain, nausea, vomiting, photophobia, and phonophobia [5]. MRM has a worse response to treatment and can easily recur. Among people under 50 years old, migraine is the primary cause of reduction of quality of life and functional impairment [6] and leads to clinical symptoms such as anxiety, depression, and insomnia [7]. Among women of reproductive age, 35%–54% suffer from MRM and report higher Migraine Disability Assessment (MIDAS) scores [8], indicating greater impairment in daily work, family life, and social participation [9]. Menstrual‐related symptoms, including MRM, cause about 9 days of annual productivity loss per person [10], which costs further add to personal and societal economic burdens.

The main therapy for MRM is staged drug treatment, including nonspecific treatment, such as nonsteroidal anti‐inflammatory drugs, nonopioid analgesics, and caffeine analgesic combinations (e.g., aspirin acetaminophen + aspirin + caffeine) for the acute phase and specific treatment for the acute phase using triptans, ergotamine, ergotamine derivatives, and gepants. For menstrual migraine (MM), short‐term prevention use of triptans or other agents may also be considered [11]. However, the therapeutic effects are not as expected due to adverse reactions such as dizziness, nausea, and gastrointestinal discomfort caused by drugs and migraine due to frequent overdoses [12]. A prospective cohort study of 500 participants showed that triptan use was associated with higher recurrence frequency and longer attack duration in patients with MRM. This suggests that increased triptan use may further elevate the risk of medication overuse [13]. Treatment of MRM has become an important public health issue, which is worth our active exploration for therapies to relieve pain in patients.

Previous studies have confirmed the clinical efficacy of acupuncture treatment for migraines, which can significantly reduce seizure frequency and the number of days with migraine [14]. In 50% of patients, the frequency of migraine is at least halved after acupuncture treatment, and the effect is more obvious than prophylactic treatment [15]. In addition, the adherence of patients receiving acupuncture treatment is better, and there is less interruption in clinical trials because of adverse reactions [16]. However, a systematic review of 826 participants showed that acupuncture was not superior to sham acupuncture in reducing monthly MM frequency, attack duration, average headache intensity, or analgesic use [17]. Although a recent systematic review reported that acupuncture reduced visual analog scale (VAS) scores, attack frequency, and duration in MM, the GRADE certainty of evidence was rated as low, and substantial heterogeneity was observed across pooled analyses [18]. Therefore, acupuncture cannot be recommended for MM until more robust evidence becomes available. In addition, reviewing the clinical evidence of treatment for MRM, there is only one monocentric RCT that found that both acupuncture and sham acupuncture can reduce MRM attacks but with no significant differences [19]. The significance of existing clinical guidance on the efficacy of electroacupuncture for MM is limited, and no definitive conclusions can be drawn [17, 20]. Consequently, high‐quality evidence‐based research is needed to validate the clinical effectiveness of electroacupuncture in treating MRM.

To further assess the clinical effectiveness and security of electroacupuncture treatment for MRM and establish an objective scientific basis, we designed a multicenter, randomized, controlled, single‐blind study. This study aimed to assess the clinical efficacy and safety of electroacupuncture in treating MRM by providing a comprehensive evaluation of migraine symptoms in patients with MRM compared with sham electroacupuncture. We hypothesized that after 12 weeks of electroacupuncture treatment, there would be a significant reduction in migraine symptoms among MRM patients, thereby decreasing their reliance on pain medication. The goal of this study was to provide robust evidence for the clinical efficacy of electroacupuncture in treating MRM and serve as a reference for developing treatment strategies for this condition.

## 2. Patients and Methods

### 2.1. Setting

**FIGURE 1:**
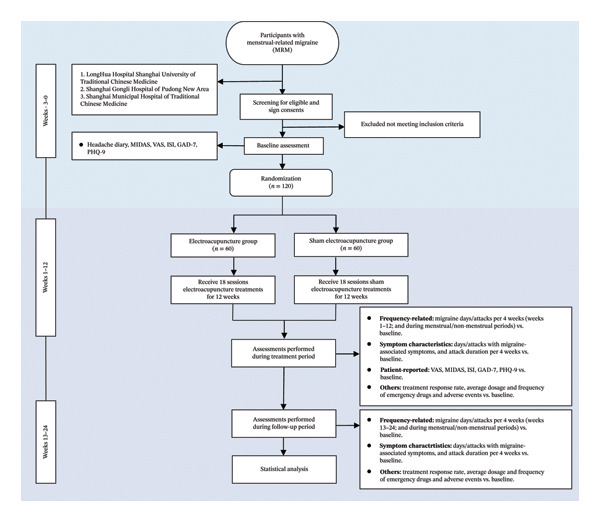
Trial flow chart. MIDAS, Migraine Disability Assessment; VAS, visual analog scale; ISI, Insomnia Severity Index; GAD‐7, Generalized Anxiety Disorder‐7; PHQ‐9, Patient Health Questionnaire‐9.

We conducted a multicenter, randomized, single‐blind, controlled clinical trial across three centers in China: LongHua Hospital Shanghai University of Traditional Chinese Medicine, Shanghai Gongli Hospital of Pudong New Area, and Shanghai Municipal Hospital of Traditional Chinese Medicine, Shanghai University of Traditional Chinese Medicine. We will recruit participants and conduct treatment in these three hospitals. Ethical approval was obtained from the Medical Ethics Committee of LongHua Hospital Shanghai University of Traditional Chinese Medicine (No. 2024LCSY150), Shanghai Gongli Hospital of Pudong New Area (GLYY1s2024‐085), and Shanghai Municipal Hospital of Traditional Chinese Medicine (2025SHL‐KY‐60‐01), and the study was registered with the International Traditional Medicine Clinical Trials Registry. A total of 120 MRM participants will be recruited for this trial and assigned to the electroacupuncture or sham electroacupuncture groups in a 1:1 ratio. The recruitment period will be from December 2024 to May 2027. A flowchart of the trial procedure is shown in Figure 1. This study adheres to the Declaration of Helsinki.

### 2.2. Participants

Participants will be eligible for inclusion if they meet the following inclusion criteria: (1) meet the diagnostic criteria for MRM without aura according to the International Classification of Headache Disorders (ICHD‐3); (2) female aged 18–50 years; (3) the courses of MRM without aura last more than 12 months [21]; (4) the frequency of migraine attack is 2–8 times on average and occurrences less than 15 days in one month within 3 months before enrollment [21]; (5) with regular menstrual cycle; and (6) agree to participate in this study and complete the signing of informed consent. Informed consent will be obtained from all participants prior to inclusion.

Participants will be excluded if they meet any of the following criteria: (1) the presence of other types of primary or secondary headache; (2) a combination of severe cardiovascular and cerebrovascular, neurologic, hematological, and acute infectious diseases; (3) active or previous history of peptic ulcer disease, gastrointestinal bleeding, or perforation; (4) use of preventive migraine medicine within the 3 months prior to the screening; (5) planned pregnancy during the treatment period, oral contraceptive distribution, or during the period of pregnancy or lactation; (6) those who had received acupuncture treatment within 1 year [22]; (7) with cardiac pacemakers; and (8) with metal allergy or serious fear of needle. All participants who fulfill the inclusion criteria and consent to join the study will sign a written informed consent form.

### 2.3. Interventions

The participants will be randomly assigned to the electroacpuncture and sham electroacupuncture groups in a 1:1 ratio. All participants will receive 6 sessions every 4 weeks (twice a week in the week before and during the menstrual period, once a week for the remaining 2 weeks), each lasting for 30 min. The participants will receive 18 sessions in total for 12 consecutive weeks and a follow‐up period of 12 weeks.

If participants had suffered from severe pain (VAS > 8 [16]), oral ibuprofen sustained‐release capsules (trade name: ibuprofen sustained‐release capsules, 300 mg, Shanghai Xinyi Tianping Pharmaceutical Co.) were allowed under the guidance of the doctor. The initial dose is 300 mg/day, and the dosage can be adjusted according to the situation; however, the maximum daily dose does not exceed 800 mg [23]. The dosage, time, and reaction to medicine will be recorded in detail in a case report form (CRF). If the patient has taken medication within the 12 h preceding the efficacy assessment, the evaluation should be postponed for a duration of 24 h.

#### 2.3.1. Electroacupuncture Group

**FIGURE 2:**
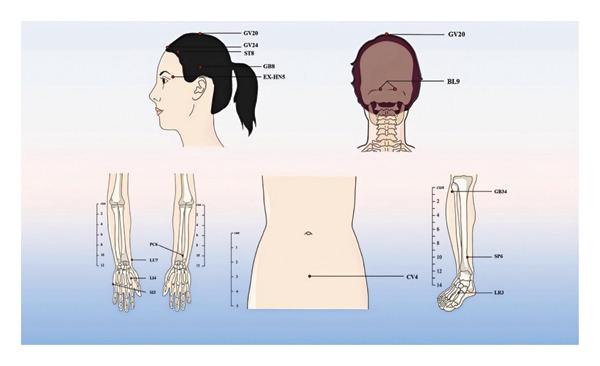
Acupoint location for the electroacupuncture and sham electroacupuncture groups. GV20: Baihui; GV24: Shenting; ST8: Touwei; GB8: Shuaigu; EX‐HN5: Taiyang; BL9: Yuzhen; LU7: Lieque; LI4: Hegu; SI3: Houxi; PC6: Neiguan; CV4: Guanyuan; GB34: Yanglingquan; SP6: Sanyinjiao; LR3: Taichong.

Participants in the electroacupuncture group will be treated with acupuncture at obligatory acupoints, including GV20, GV24, CV4, EX‐HN5 (bilateral), LU7 (bilateral), and SP6 (bilateral). According to the meridian diagnosis and the location of the headache in the acute attack, additional acupoints will be selected: LI4 (bilateral) and ST8 (bilateral) for Yangming migraine (pain predominantly in the forehead, supraorbital ridge, and nasal root); LR3 (bilateral) and PC6 (bilateral) for Jueyin migraine (pain predominantly at the vertex of the head); GB8 (bilateral) and GB34 (bilateral) for Shaoyang migraine (pain predominantly on the side of the head); and SI3 (bilateral) and BL9 (bilateral) for Taiyang migraine (pain predominantly in the occipital region or radiating downward to the nape). Details of the acupoint position and acupuncture technique are shown in Table 1 and Figure 2. (Acupoint location: refer to The Location of Acupoints: State Standard of the People’s Republic of China [GB/T 12346–2021]). Participants will be placed in the supine position, and all acupoints will be routinely disinfected. The acupoints will be treated with balanced tonification and purging manipulation to achieve the Deqi sensation with feelings of soreness, numbness, heaviness, and distension through techniques such as lifting, thrusting, and twisting. A disposable acupuncture needle (Suzhou Medical Supplies Factory Co., Hua Tuo, China) will be selected as the acupuncture tool. Electroacupuncture treatment will be conducted in the form of dense and sparse waves using the HANS acupoint nerve stimulator (model LH 200A; Nanjing Jisheng Medical Technology Co. Nanjing, China), connecting GV20 to the left EX‐HN5 and GV24 to the right EX‐HN5. The electroacupuncture frequency was set at 2/100 Hz, with intensity adjusted to the patient’s comfort [24].

**TABLE 1:** The location and manipulation of acupoints in the electroacupuncture group.

Acupoint	Location	Manipulation
GV20 (Baihui)	On the anterior midline of the head. This acupoint is found 5 cun superior to the anterior hairline.	The needle is inserted horizontally by 0.5–0.8 cun.
GV24 (Shenting)	On the anterior midline of the head. This acupoint is found 0.5 cun superior to the anterior hairline.	The needle is inserted horizontally by 0.3–0.5 cun.
CV4 (Guanyuan)	On the anterior midline of the abdomen. This acupoint is found 3 cun inferior to the center of the umbilicus.	The needle is inserted vertically by 1.0–2.0 cun.
EX‐HN5 (Taiyang)	On the lateral face of the head. This acupoint is found in the depression one fingerbreadth (middle finger) posterior to the midpoint between the medial end of the eyebrow and the lateral canthus of the eye.	The needle is inserted vertically by 0.3–0.5 cun.
LU7 (Lieque)	On the lateral side of the forearm. This acupoint is found 1.5 cun superior to the palmar wrist crease, between the tendons of the abductor pollicis longus and the extensor pollicis brevis muscles, in the groove for the abductor pollicis longus tendon.	The needle is inserted obliquely upward by 0.3–0.5 cun.
SP6 (Sanyinjiao)	On the medial aspect of the leg. This acupoint is found 3 cun above the medial malleolus, at the back edge of the tibia.	The needle is inserted vertically by 1.0–1.5 cun.
LI4 (Hegu)	On the dorsum of hand. This acupoint is found radial to the midpoint of the second metacarpal bone.	The needle is inserted vertically by 0.5–1.0 cun.
ST8 (Touwei)	On the head. This acupoint is found 0.5 cun directly superior to the anterior hairline at the corner of the forehead, 4.5 cun lateral to the anterior median line.	The needle is inserted horizontally by 0.5–0.8 cun.
LR3 (Taichong)	On the dorsum of the foot. This acupoint is found between the first and second metatarsal bones, in the depression distal to the junction of the bases of the two bones, over the dorsal pedal artery.	The needle is inserted vertically by 0.5–1.0 cun.
PC 6 (Neiguan)	On the inner side of the forearm. This acupoint is 2 cun above the wrist crease, positioned between the tendons of the palmaris longus and flexor carpi radialis.	The needle is inserted vertically by 0.5–1.0 cun.
GB8 (Shuaigu)	On the head directly superior to the auricular apex. This acupoint is found 1.5 cun superior to the temporal hairline.	The needle is inserted horizontally by 0.5–0.8 cun.
GB34 (Yanglingquan)	On the fibular aspect of the leg. This acupoint is found in the depression anterior and distal to the head of the fibula.	The needle is inserted vertically by 1.0–1.5 cun.
SI3 (Houxi)	On the dorsum of the hand. This acupoint is found in the depression proximal to the ulnar side of the fifth metacarpophalangeal joint, at the border between the red and white flesh.	The needle is inserted vertically by 0.5–1.0 cun.
BL9 (Yuzhen)	On the head. This acupoint is found 1.3 cun lateral to the posterior median line, at the same level as the external occipital protuberance.	The needle is inserted horizontally by 0.3–0.5 cun.

#### 2.3.2. Sham Electroacupuncture Group

Participants in the sham electroacupuncture group will be treated with sham acupuncture at the same acupoints as those in the electroacupuncture group. To fix the needle, the adhesive pads will be attached to the acupoints, and blunt needles without a sharp tip (Guizhou Andi Medical Equipment Co.) were passed through the pad to reach the skin surface, without skin breakage or Deqi sensation. The sham acupuncture device used above has been patented by the China National Intellectual Property Administration (Patent Number: ZL 2023 2 0605629.3). Additionally, a specialized power line that is disrupted with a normal appearance (the electric needle instrument shows the on state, but it is not actually powered on) connects the same acupoints as those in the electroacupuncture group. The needle retention time, treatment frequency, and treatment duration will be the same as those in the electroacupuncture group.

## 3. Outcomes

At baseline, basic information about the participants will be collected, including age, body mass index (BMI), duration of migraine, accompanying symptoms of migraine (nausea, vomiting, photophobia, and phonophobia), and medication history. A 28‐week headache diary needs to be kept (including a 4‐week baseline period, 12‐week treatment period, and 12‐week follow‐up period). The headache diary will record daily frequency, time, location, VAS score, and associated symptoms of migraine as well as medications taken and menstrual periods. The schedule of enrollment and assessment is shown in Table 2.

**TABLE 2:** Schedule of enrollment, interventions and assessments.

Study period		Enrollment	Intervention period			Follow‐up period
Time point		Weeks −3–0	Weeks 1–4	Weeks 5–8	Weeks 9–12	Weeks 13–24

Eligibility screening		×				

Sign informed consent		×				

Medical history		×				

Randomization		×				

Intervention						

Electroacupuncture			×	×	×	

Sham electroacupuncture			×	×	×	

Primary outcomes (vs. baseline)						

Migraine days and attacks during Weeks 9–12					×	

Secondary outcomes (vs. baseline)						

Frequency‐related	Migraine days/attacks per 4 weeks (Weeks 1–24, excluding Weeks 9–12)		×	×		×
	Migraine days/attacks per 4 weeks (during menstrual/nonmenstrual periods)	×	×	×	×	×

Symptom characteristics	Days/attacks with migraine‐associated symptoms, and attack duration per 4 weeks	×	×	×	×	×

Patient‐reported	VAS	×	×	×	×	×
	MIDAS	×			×	
	ISI	×			×	
	GAD‐7	×			×	
	PHQ‐9	×			×	

Others	Treatment response rate (the proportion of patients with ≥ 50%, 75%, or 100% reduction per four weeks of migraine days)		×	×	×	×
	Average dosage and frequency of emergency drugs	×	×	×	×	×

Adverse events			×	×	×	×

Success of blinding					×	

### 3.1. Primary Outcome

The primary outcome was migraine days and attacks during Weeks 9–12 vs. baseline. Data were recorded in a headache diary.

### 3.2. Secondary Outcomes (All vs. Baseline Unless Specified)

The secondary outcomes included the following:

1. Frequency‐related: migraine days/attacks per 4 weeks (Weeks 1–24, excluding Weeks 9–12; and during menstrual/nonmenstrual periods).
2. Symptom characteristics: days/attacks with migraine‐associated symptoms (nausea, vomiting, photophobia, and phonophobia) and attack duration per 4 weeks.
3. Patient‐reported: (a) VAS: The VAS score is derived from the headache diary, which will be evaluated during the 4‐week baseline period and Weeks 1–24. (b) MIDAS [25]: This is a questionnaire used to assess the degree of migraine‐related disability and reflect the severity of migraine. The questionnaire is simple and effective and has been widely recognized and accepted by scholars worldwide. It was translated into Chinese in 2006 and has good test–retest reliability and validity. This will be evaluated at Weeks 0 and 12. (c) Insomnia Severity Index (ISI) [26]: It evaluates the severity of subjective insomnia in patients over the past week, including seven items, each with 0–4 points. The higher the score, the more serious the degree of insomnia. This will be evaluated at Weeks 0 and 12. (d) Generalized Anxiety Disorder‐7 (GAD‐7) (Copyright 1999 Pfizer Inc., all rights reserved) [27]: This scale is commonly used to evaluate generalized anxiety disorder. The scale evaluates the frequency and severity of anxiety symptoms in the past 2 weeks through seven questions. This will be evaluated at Weeks 0 and 12. (e) Patient Health Questionnaire‐9 (PHQ‐9) (Copyright 1999 Pfizer Inc., all rights reserved) [27]: It is a simple and effective self‐rating scale for assessing whether individuals have depressive symptoms and their severity. It consists of nine questions covering common symptoms of depression. This will be evaluated at Weeks 0 and 12.
4. Others: treatment response rate (the proportion of patients with a reduction of ≥ 50%, 75%, or 100% per four weeks migraine days compared with baseline [28]), average dosage, and frequency of emergency drugs.

The above data regarding MRM days, attacks, duration, associated symptoms, VAS scores, and usage of remedial drugs were all derived from the headache diary. The participants will be required to complete a headache diary for at least 20 of the 28 days [29].

### 3.3. Safety Evaluation

Adverse events (AEs) due to electroacupuncture treatment include the following: (1) possible adverse electroacupuncture treatment reactions, such as ecchymosis, fainting, and pain. (2) Aggravation due to excessive acupuncture stimulation. Researchers will classify AEs into three levels based on their severity: mild, moderate, and severe or medically significant. Researchers will evaluate these AE data from the perspectives of severity and causality. All AEs will be recorded in the CRF in detail, including severity, duration, relationship with research and treatment, severity grade, treatment, and outcome. Once any serious AE occurs, we will discontinue the participant’s treatment and decide whether to continue the study.

## 4. Sample Size Calculation

This study was a multicenter, randomized, single‐blind, controlled clinical trial, in which the primary outcome was migraine days and attacks during Weeks 9–12 vs. baseline. According to the preliminary test results derived from an internal, unpublished pilot observation conducted by our research team between April 2024 and August 2024, which involved 15 participants in each group, of the research group, the mean and standard deviation of migraine days in the electroacupuncture group and the sham electroacupuncture group were 3.13 ± 1.77 and 1.98 ± 1.46, respectively. The mean and standard deviation of migraine attacks in the electroacupuncture group and sham electroacupuncture group were 2.13 ± 1.12 and 1.17 ± 1.20, respectively. Setting the ratio of the electroacupuncture group to the sham electroacupuncture group as 1:1, the test level *α* = 0.05 (bilateral), and power (1−*β*) = 0.90, the sample size of each group was calculated to be 51 cases. Considering a shedding rate of 15%, 60 cases will be taken in each group and 120 cases in total.

## 5. Randomization

The study will use the block randomization method. Each center competes to be included in the group. The random scheme for the study is generated by the “Proc plan” program of SAS 9.4 statistical analysis software, and the random distribution cards are made, which are sealed with an opaque envelope. The envelope serial number is the same as that of the card. The envelope will be opened in the order of visits by participants. The corresponding researchers at each center will randomly assign participants into two groups based on the contents of the envelope card in a 1:1 ratio: eligible participants to the electroacupuncture group or the sham electroacupuncture group.

## 6. Blinding

Participants, efficacy evaluators, data analysts, and statistical analysts will not be aware of the grouping status of the subjects, in addition to the acupuncturists. To ensure the success of blinding, participants will be requested to restrict communication of the treatment condition and visit the hospital according to the appointment. Patients will receive treatment in the supine position at independent clinics or with screens. All participants will be requested to wear eye masks to avoid seeing the needles visually. Similarly, evaluators who do not know the grouping status will evaluate and record the observed indices. Statistical analysis using the blind method will be performed by a third party to ensure the authenticity of the research results. In addition, all researchers will receive professional training about the specifications of the research and be requested to maintain the separation of departments. In addition, the questionnaire will be administered after treatment to evaluate blinding. Participants will need to answer the question of “what kind of treatment do you think you have received?” and they will choose the answer from “electroacupuncture, sham electroacupuncture, and unclear. Bang’s Blinding Index will be used for blind evaluation.

## 7. Analysis

Data collection and entry for this study will be conducted by specially trained clinical staff. Efficacy analyses will be performed on both the intention‐to‐treat (ITT) population and the per‐protocol set (PPS) population. Missing values for patients in each group will be imputed using the last observation carried forward (LOCF) method. The safety analysis set (SAS) will be analyzed within the ITT population, which includes all randomized subjects who received at least one treatment.

Given that additional acupoints are selected based on the specific location of headache during acute attacks, we will quantitatively report the percentage of patients in each subgroup and compare baseline characteristics and changes in primary outcomes across subgroups.

SAS software (Version 9.4; SAS Institute Inc., Cary, North Carolina 27513, US) will be used for the statistical analysis. Qualitative data will be described as cases and percentages, and quantitative variables will be described as mean, median, standard deviation, and quartile range. Statistical significance was set at *p* < 0.05.

The primary outcome of this study was migraine days and attacks during Weeks 9–12 vs. baseline. The data will be recorded in a headache diary and analyzed using two independent sample *t*‐tests or rank‐sum tests. For the analysis of secondary outcomes, two independent sample *t*‐tests or rank‐sum tests were used for quantitative data, and the chi‐square test or Fisher’s exact test will be used for qualitative data. Data obtained from repeated measurements will be statistically analyzed using generalized estimating equations. The chi‐square test will also be used for a safety indicator.

The headache diary in this study involved multiple data collections. If participants complete the headache diary for at least 28 days in consecutive 4‐week periods, researchers will directly calculate the number of migraine days per month. If the number of days that participants complete the headache diary in consecutive 4 weeks is less than 20, monthly migraine days will be classified as missing. Missing days will be defined as days in which participants do not complete the evening report and do not report headaches. Estimation over 4 weeks will be used for missing data, using the number of migraine days within four weeks divided by the number of days observed during four weeks multiplied by 28 as the monthly score for the four weeks.

## 8. Data Collection, Management, and Monitoring

In this study, the designated researchers will use the preestablished CRF to collect the participants’ basic information and clinical data. Prior to the clinical trial, all personnel involved in data collection will receive standardized training on data collection, entry, and management. The double‐entry method will be used to ensure data accuracy. To avoid discrepancies, the data will be entered independently by two researchers, followed by data verification. Besides, a data safety monitoring team has been established for the trial’s performance and safety, which is mainly composed of experts from mainland China. Data management follows the specified schedule, procedures, and guidelines outlined in the data management plan. The CRF, informed consent, and other materials will be archived by the LongHua Hospital Shanghai University of Traditional Chinese Medicine.

## 9. Discussion

MRM is characterized by intricate physiological and pathological mechanisms, resulting in more severe clinical manifestations and prolonged duration compared to non‐MRM. The cyclic recurrence of MRM increases patients’ reliance on analgesic medications, possibly increasing their susceptibility to drug tolerance [30]. This recurrent nature significantly affects patients’ physical and mental health [7], diminishes their quality of life [6], and imposes economic burden due to missed work [31]. Consequently, the implementation of prevention and treatment strategies is critical to improve the prognosis of patients with MRM. However, the adverse reactions associated with acute and preventive pharmacotherapy, along with the risk of overdose migraines due to frequent medication use, render long‐term drug therapy a concern that cannot be overlooked [12]. Therefore, there is an urgent need to develop safe and effective alternative treatment options.

The clinical efficacy of acupuncture in the treatment of migraine has been substantiated in numerous RCTs [32]; however, related research is limited regarding its effectiveness, specifically for MRM. This study was designed to address the unique characteristics of MRM and proposes an EA intervention. MRM is closely linked to the menstrual cycle, particularly during premenstrual and menstrual phases. Accordingly, our intervention included two treatments in the premenstrual and menstrual weeks, along with one treatment in each of the other 2 weeks. This approach not only enhances the specificity of treatment but also reduces the time burden on patients. The location of migraine attacks is another critical factor in electroacupuncture treatment. Thus, during acute migraine phases, supplementary acupoints are chosen based on the location of pain to optimize treatment effectiveness. In addition to acupoint selection, the choice of electrical stimulation parameters is equally important. Previous studies have shown that alternating stimulation at 2 and 100 Hz using a sparse‐dense wave can simultaneously activate the enkephalin and dynorphin systems, producing a stronger synergistic analgesic effect than single‐frequency stimulation [33]. Furthermore, a randomized controlled trial involving 90 patients with migraine confirmed that variable‐frequency (2/100 Hz) electroacupuncture exhibited superior clinical efficacy compared with low‐frequency (2 Hz) or high‐frequency (100 Hz) electroacupuncture in the treatment of migraine [34]. Taken together, these findings provide a solid physiological and clinical rationale for using the 2/100 Hz alternating sparse‐dense wave protocol in the present trial. Furthermore, to bolster the reliability of our study, both electroacupuncture and sham electroacupuncture treatments will utilize the same auxiliary device (patent number: ZL 2023 20605629.3). This strategy not only facilitates blinding but also encourages patient adherence to treatment. Previous clinical studies have confirmed the feasibility of placebo application [35].

However, this study had certain limitations. To adhere to medical ethical standards, the patients were permitted to use emergency medications during the study period, which may have influenced the assessment of therapeutic efficacy. To mitigate this impact, we conducted subgroup and covariate analyses. Additionally, because of the unique nature of acupuncture techniques, the acupuncturists could not be blinded in this study.

In conclusion, high‐quality clinical studies investigating the efficacy and safety of electroacupuncture for MRM are essential. We anticipate that the results will provide clinical evidence for the use of electroacupuncture in the treatment of MRM, ultimately enhancing the clinical symptoms and quality of life of patients.

## Author Contributions

Shihan Jiang, Qing Ye, and Sichun Gu contributed equally to this article as co‐first authors. Lumin Liu, Qing Ye, and Ping Yin contributed to the study design. Shihan Jiang, Lumin Liu, and Ping Yin drafted the manuscript. Qiang Li and Qi Jin completed the revision of manuscripts. Zhicheng Guo, Lumin Liu, and Shihan Jiang were responsible for acupuncture treatment in the trial. Qiang Li and Qi Jin participated in trial communication and monitoring. Sichun Gu was responsible for data collection. All authors participated in the reading, discussion, and revision of the manuscript.

## Funding

This work was supported by the Shanghai Municipal Health Commission Health Industry Clinical Research Special Top Project (202340110), Construction of Traditional Chinese Medicine Inheritance and Innovation Development Demonstration Pilot Projects in Pudong New Area‐High‐Level Research‐Oriented Traditional Chinese Medicine Hospital Construction (YC‐2023‐0901), awarded to Ping Yin, and Shanghai Shenkang Hospital Development Center (SHDC2023CRD005), awarded to Qing Ye.

## Disclosure

All authors approved the protocol for publication.

## Conflicts of Interest

The authors declare no conflicts of interest.

## Patient Perspective

Patients and/or the public were not involved in the design, conduct, reporting, or dissemination plans of our research.

## Data Availability

Data sharing does not apply to this article as no datasets were generated or analyzed during the current study.
